# Molecular profile of a pleomorphic adenoma of the hard palate: A case report

**DOI:** 10.1097/MD.0000000000021207

**Published:** 2020-07-17

**Authors:** Yoshiyuki Iida, Masakuni Serizawa, Takashi Mukaigawa, Tomoyuki Kamijo, Takashi Nakajima, Koiku Asakura, Masatoshi Kusuhara, Ken Yamaguchi, Tetsuro Onitsuka

**Affiliations:** a Division of Head and Neck Surgery, Shizuoka Cancer Center Hospital; b Drug Discovery and Development Division, Shizuoka Cancer Center Research Institute; c Division of Pathology; d Division of Diagnostic Radiology, Shizuoka Cancer Center Hospital; e Shizuoka cancer center, Shizuoka, Japan.

**Keywords:** fusion gene, hard palate, next generation sequencing, *PLAG1*, pleomorphic adenoma

## Abstract

**Rationale:**

Pleomorphic adenoma (PA) is the most common benign tumor of salivary glands. PAs have the potential for regional and distant metastases that preserve their benign phenotype; they also have the potential for malignant transformation. The molecular pathogenesis of malignant neoplasms has been studied extensively in recent years, unlike that of benign tumors, such as PA.

**Patient concerns:**

In this case report, we identified the molecular signatures of a 57-year-old Japanese woman. Our patient presented with a swelling of the hard palate with an erosive appearance.

**Diagnoses:**

The patient was diagnosed with a right hard palate tumor suspected to be a malignant neoplasm.

**Interventions:**

Partial maxillary resection and reconstruction were performed.

**Outcomes:**

There was no obstacle to swallowing or dysarthria after surgery. There was no sign of recurrent palatal tumor 4 years after the operation. Using next generation sequencing, 5 nonsynonymous mutations and *CHCHD7-PLAG1* fusion genes were detected. Moreover, gene expression profiling indicated the possibility of the activation of several cancer-related signaling pathways. Although the *PLAG1* gene is predicted to play a crucial role in PA tumorigenesis, its over-expression is reported to mediate multiple downstream factors. In this case, various up- and downregulated RNA signaling pathways, including MAP kinase signaling, PI3K/AKT1/MTOR signaling, JAK/STAT signaling, and PD-L1 signaling, were revealed.

**Lessons:**

These molecular profiles of PA may elucidate the mechanism of metastasis, preserving its benign phenotype and carcinoma ex PA.

## 1 Introduction

Recent progress in the molecular profiling of malignant neoplasms has improved our understanding of the molecular pathogenesis of many cancer types and also contributed to the development of novel treatment strategies. However, the elucidation of the molecular signatures of benign tumors, such as pleomorphic adenoma (PA), has been inadequate. PA is the most common benign type of salivary gland tumors, accounting for 60% of all salivary gland tumors. Fusion genes, such as *PLAG1*^[1,2]^ and *HMGA2,*^[3,4,5]^ have been reported to be involved in its pathogenesis. PA has clinical features, such as distant metastases and malignant transformation, which are inconsistent with the benign phenotype in local sites. However, the molecular mechanisms underlying these inconsistent clinical features have not been elucidated. In this case, we identified the molecular signatures of a patient with PA using next-generation sequencing and gene expression profiling.

## 2 Case presentation

This is a case that is enrolled in project high-tech omics-based patient evaluation^[6]^ a study launched at Shizuoka Cancer Center to evaluate the biological characteristics of cancer and diathesis of each patient by multiomics-based analyses. Ethical approval was granted by the institutional review board (Authorization Number: 25–33). Written informed consent from the patient was obtained for the publication of this case report.

**Figure 1:**
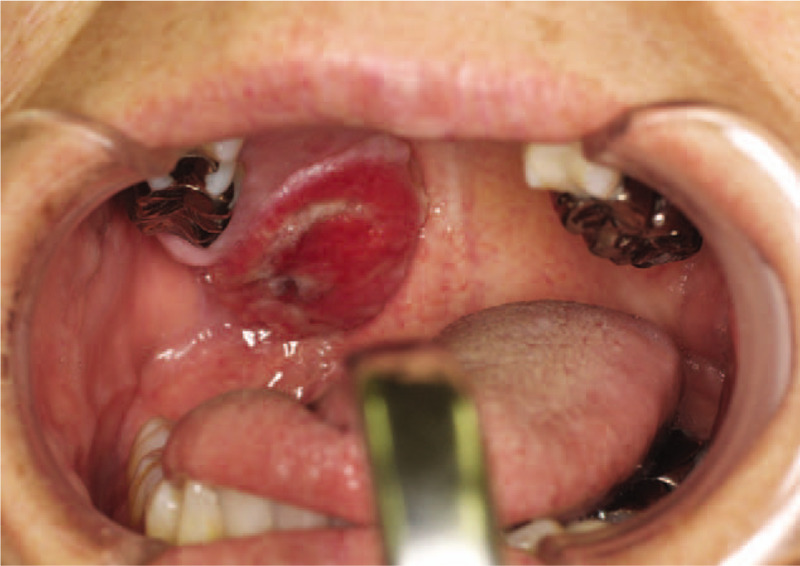
The lesion had a size of 21 mm × 20 mm with everted edges and an ulcerative surface.

**Figure 2:**
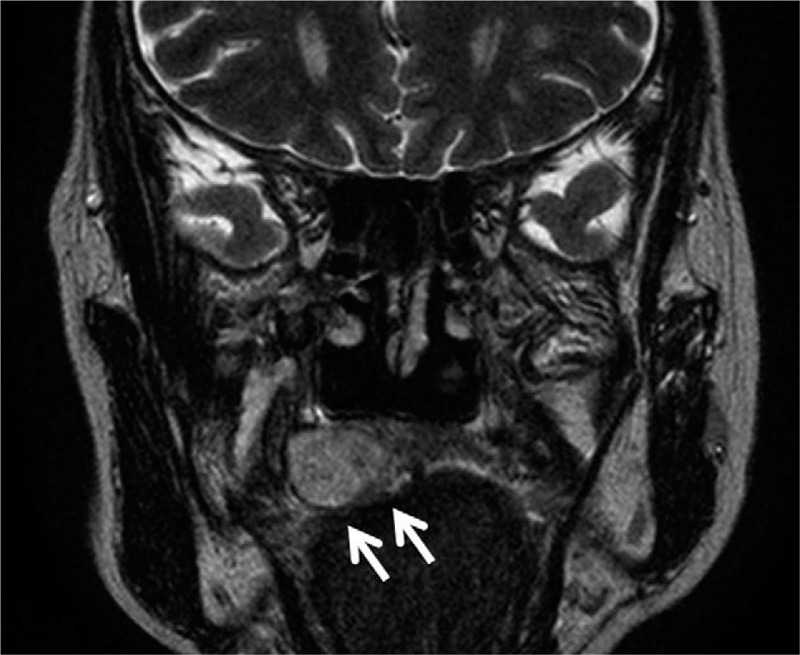
A well-defined oval lesion in the right side of the palate expansive to a maxilla bone.

A 57-year-old Japanese woman presented to a hospital with a 2-month-old (or possibly longer) indolent oral swelling. A previous clinical examination at another facility reported the presence of a submucosal mass on the right side of her hard palate. After the biopsy, the palatal swelling acquired an erosive appearance, and she was referred to our hospital. She had a medical history of hepatitis C infection. Since she was a non-smoker with occasional social alcohol consumption, the risk factors were minimal. Upon clinical examination, the lesion measured 21 mm × 20 mm with everted edges and had an ulcerative surface (Fig. 1). The tumor was non-mobile; the adjacent mucosa was normal in color. The axial and coronal reconstruction images of the palatal swelling were obtained, using computed tomography and evaluated. A tumor, which extended over the maxilla bone, was in the left side of the palate. Magnetic resonance imaging revealed a well-defined oval lesion (Fig. 2). No significant regional lymph node enlargement was observed. The biopsy specimen showed a prominent hyperplastic downward growth of the covering squamous epithelia, which suggested the presence of a malignant neoplasm. Additional biopsy specimens were similar in description to the initial specimen.

Under general anesthesia with orotracheal intubation, partial maxillary resection was performed within adequate surgical margin. After the tumor was removed, reconstruction of the walls and floor of the bony cavity was done using a vascularized anterolateral thigh flap, and concurrently performed prophylactic neck dissection (level I-III). No obstacle to swallowing or dysarthria was observed after surgery. No sign of recurrent palatal tumor 4 years after the operation was detected.

**Figure 3:**
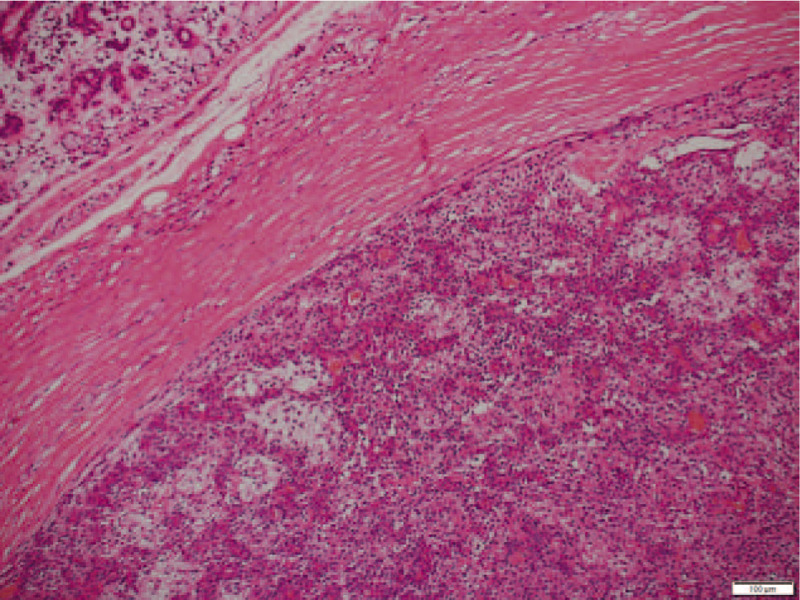
The tumor was composed of a mixture of epithelial and fibromyxoid components. The epithelial component was glandular in nature and composed of double-layered cells and frequent foci of squamous metaplasia. In the fibromyxoid component, spindle- or stellate-shaped myoepithelial cells were loosely present and intimately related to outer-layered cells of the epithelial component.

The tumor was located between the oral squamous epithelia and minor salivary glands and surrounded with a thick fibrous capsule. At the oral mucosal site, the fibrous capsule had been destroyed by chronic inflammation; the tumor border was unclear and intermingled with granulation and fibrotic tissues. Histologically, the tumor was composed of a mixture of epithelial and fibromyxoid components. The epithelial components were glandular and composed of double-layered cells and frequent foci of squamous metaplasia. In the fibromyxoid components, spindle- or stellate-shaped myoepithelial cells were loosely present and intimately related to outer-layered cells of the epithelial components. The tumor showed slight cellular atypia with no necrosis and abnormal mitosis. No lymph node metastasis was detected. The tumor was finally diagnosed as an ulcerated PA (Fig. 3).

Whole exome sequencing to identify tumor-specific genetic alterations was performed on an Ion Proton system using an Ion Torrent AmpliSeq RDY Exome Kit (Thermo Fisher Scientific, Massachusetts, USA) according to the manufacturer's instructions. The corresponding peripheral blood sample was used as a control. Tumor mutation burden (mutations/Mbase) was 0.34. A total of 11 mutations, including 5 nonsynonymous mutations, 2 synonymous mutations, and 4 mutations located in non-coding regions, were detected. All gene symbols are referred from the My Cancer Genome website.^[7]^ The nonsynonymous mutations included 1 frameshift mutation in the *ARHGAP9* gene and 4 missense mutations in the *USF3*, *SOHLH1*, *SALL3,* and *FAM109B* genes (Table 1). Significant copy number alterations were not observed. A *CHCHD7-PLAG1* fusion gene (COSMIC ID, COSF1087) was detected in an Ion Proton system (Thermo Fisher Scientific) using an original targeted RNA-sequencing panel, which was designed to detect transcripts of 491 fusion genes based on the Ion AmpliSeq RNA Fusion workflow (Thermo Fisher Scientific), as described in a previous report.^[8]^

**Table 1:**
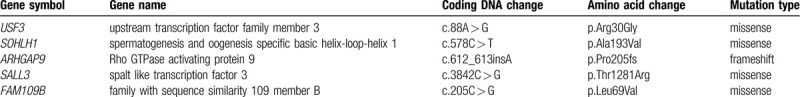
Detected nonsynonymous mutations.

Gene expression profiling was performed using the SurePrint G3 Human Gene Expression 8 × 60K v2 Microarray (Agilent Technologies) according to the manufacturer's instructions. Up- (Log_2_ ratio of tumor to healthy tissue ≥ 2) and down-regulated (Log_2_ ratio of tumor to healthy tissue ≤ -2) genes in tumor cells, compared with adjacent healthy tissues, were 1,064 and 829, respectively. Expression of *PLAG1,* which is a partner gene of the detected *CHCHD7-PLAG1* fusion gene, was strongly upregulated in the tumor (log_2_ ratio, 4.2). To clarify the association between detected expression abnormalities and tumorigenesis, we focused on genes annotated in OncoKB and associated with solid cancer.^[7]^ Pathways were classified based on classifications in previous reports.^[9,10,11]^ A total of 38 up- and 18 down-regulated genes in the tumor, compared with adjacent normal tissues, were included. Among the 18 down-regulated genes, 2 genes (*EPCAM* and *TMPRSS2*) not assigned to cancer-related pathways were excluded from analysis, and the remaining 54 genes listed in Table 2 were classified by each pathway.

**Table 2:**
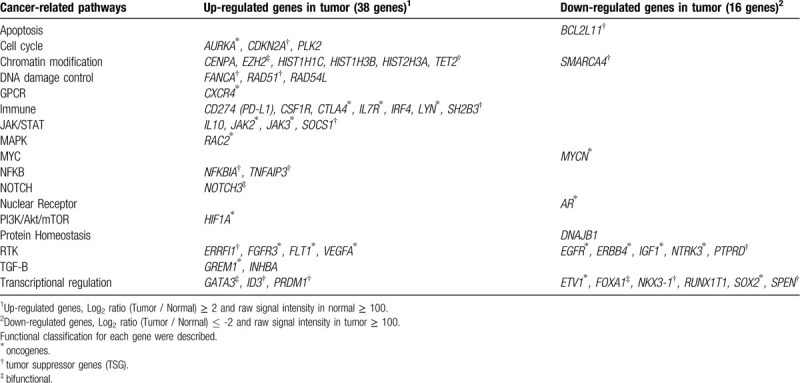
Cancer-related genes which showed tumor-specific gene expression abnormalities.

## 3 Discussion

The main treatment for PA is complete surgical excision with adequate margins. Although PA is a benign tumor, surgical manipulation and incomplete removal of the tumor have been suggested to have the potential to lead to tumor cell dislodgement and vascular implantation, and eventually a hematogenous spread.^[12]^ When metastasis of PA occurs, its benign phenotype is preserved on rare occasions; in which case, malignant transformation is mostly a further clinical problem. Carcinoma ex PA is a malignant tumor of the salivary gland that arises from benign PA and is universally regarded as high-grade cancer.

Five nonsynonymous mutations detected in whole exome sequencing are variants of unknown significance, for which no association with cancer has been reported. To evaluate the biological associations between genes in which those mutations were detected and PA, we need to identify whether mutations in those genes are detected in other patients with PA using archived specimens and by further molecular profiling studies of PA. The feature, in this case, is that the *PLAG1* fusion gene has been identified. Fusion genes have been discovered in various diseases, including hematological malignancies, sarcomas, and solid cancers. Over 20,000 fusion genes have been identified currently.^[13]^ Historically, karyotypic alterations of parotid PAs have been studied since 1973.^[14]^ An extensive study of 220 PAs found that 50% had normal karyotypes, 25% had 8q12 rearrangements, and 13.2% had 12q13–15 rearrangements.^[15]^ In 1997, Kas et al^[1]^ reported *PLAG1–CTNNB1* fusion as a critical event in the tumorigenesis of PA and CHCHD7,^[2]^ LIFR,^[16]^ and TCEA1^[2,4]^ as fusion partners of *PLAG1*. Subsequently, *HMGA2*-*NFIB*,^[2]^*HMGA2*-*FHIT*,^[3]^ and *HMGA2*-*WIF1*^[5]^ in PAs have also been reported. In the recent report of 105 and 11 cases of PAs and carcinoma ex PA, respectively, approximately 40% of cases harbored *PLAG1* fusion genes: *CTNNB1-PLAG1* in 22 cases, *CHCHD7-PLAG1* in 14 cases, *LIFR - PLAG1* in 4 cases, and *HMGA2* fusion genes in 2 cases.^[16]^ Hence, the *PLAG1* gene is predicted to play a crucial part in the tumorigenesis of PA. Conversely, approximately half of PAs do not undergo chromosomal rearrangements.^[17]^ In 1 compelling case of distant metastasis, Akiba et al found that both the primary PA and metastasis sites were positive for PLAG1 protein, but neither tumor harbored the *PLAG1* fusion gene.^[18]^

Gene expression pattern revealed a distinct expression signature consisting of various up- and downregulated transcripts. CD274 (PD-L1), exploited by tumors to attenuate anti-tumor immunity, was strongly upregulated in the tumor (log_2_ ratio, 4.1), suggesting that anti-PD-1/PD-L1 therapy might be sufficient for this patient. Observed upregulation of the well-known angiogenetic factor, VEGFA, (log2 ratio, 2.3) might be caused by upregulation of upstream factors, such as HIF1A, which play roles as transcriptional regulators of VEGFA^[19]^ and some receptor tyrosine kinases (RTKs). Significant expression abnormalities were detected in many genes included in well-known several cancer-related pathways, such as RTKs, Mitogen-activated protein (MAP) kinase, Janus kinase (JAK)/signal transducer and activator of transcription (STAT), and PI3K/AKT/mTOR. This suggests the possibility that these observed phenomena might contribute to clinical characteristics of PA, such as distant metastases and malignant transformation, which are inconsistent with the benign phenotype in its local site.

Overexpression of *PLAG1* was reported to mediate multiple downstream factors, including the insulin-like growth factor (IGF). In this tumor, expression of IGF-I was strongly downregulated (log_2_ ratio, -3.3). IGF-I and IGF-II are multifunctional regulatory peptides that primarily mediate proliferation, differentiation, and survival effects. *In vivo*, the WNT signaling pathway was previously shown to be activated in transgenic mice overexpressing PLAG1.^[20]^ As described above, PA is benign but has various expression changes, which may be a factor in the development of malignancy and metastasis.

## 4 Conclusions

Although we obtained molecular data on PA in this case, the pathogenesis of PA remains mostly unclear. This data may serve as a theoretical framework for further research into the development of novel treatment strategies for surgically untreatable distant metastases or malignant transformations. More in-depth multiomics studies with large number of cases are required to further ascertain the tumorigenesis of PA. However, we believe that data from our patient highlight the possibility that targeted therapy could be developed to induce dormancy in PAs to prevent their distant metastases and malignant transformation.

## Acknowledgments

We thank the staff at Shizuoka Cancer Center Hospital for their invaluable help.

## Author contributions

**Data curation:** Takashi Nakajima, Koiku Asakura

**Investigation:** Masakuni Serizawa

**Project administration:** Ken Yamaguchi, Masatoshi Kusuhara

**Resources:** Takashi Nakajima, Tomoyuki Kamijo,

**Supervision:** Tetsuro Onitsuka

**Writing – original draft:** Masakuni Serizawa

**Writing – review & editing:** Yoshiyuki Iida
